# Clinical outcomes and reintervention after endoscopic retrograde cholangiopancreatography in primary sclerosing cholangitis in absence of cholangitis

**DOI:** 10.1007/s12664-024-01630-1

**Published:** 2024-07-12

**Authors:** Ryosuke Horio, Jun Kato, Takashi Taida, Yuki Ohta, Keiko Saito, Yuhei Oyama, Hayato Nakazawa, Yukiyo Mamiya, Chihiro Goto, Satsuki Takahashi, Mayu Ouchi, Akane Kurosugi, Michiko Sonoda, Motoyasu Kan, Tatsuya Kaneko, Hiroki Nagashima, Naoki Akizue, Koji Takahashi, Kenichiro Okimoto, Hiroshi Ohyama, Tomoaki Matsumura, Izumi Ohno, Naoya Kato

**Affiliations:** Department of Gastroenterology, Graduate School of Medicine, Chiba University, 1-8-1 Inohana, Chuo-Ku, Chiba, 260-8670 Japan

**Keywords:** Cholangiocarcinoma, Cholangitis, Endoscopic retrograde cholangiopancreatography, Magnetic resonance cholangiopancreatography, Primary sclerosing cholangitis

## Abstract

**Background and Aim:**

Endoscopic retrograde cholangiopancreatography (ERCP) may help detect cholangiocarcinoma in patients with primary sclerosing cholangitis (PSC), but it may be associated with complications. This study was aimed at determining the prognostic impact of ERCP on patients with PSC without cholangitis.

**Methods:**

Patients with PSC without cholangitis were divided into two groups: those who underwent ERCP within three years after diagnosis (ERCP-performed group) and those who did not (non-ERCP group). These groups were compared in terms of clinical outcomes (liver-related death or liver transplantation, endoscopic treatment requirement and repeated cholangitis) and the composite outcome.

**Results:**

Of 99 patients with PSC with detailed medical history, 49 were included in the ERCP-performed group and 21 in the non-ERCP group. In Kaplan-Meier analysis, the non-ERCP group was less likely to achieve the three outcomes and the composite outcome, showing statistical significance (endoscopic treatment requirement; *p* = 0.017 and composite outcome; *p* = 0.014). A Cox proportional hazards model indicated that ERCP in the asymptomatic state was a significant predictor of endoscopic treatment requirement (hazard ratio [HR]: 4.37, 95% confidence interval [CI]: 1.03–18.59) and the composite outcome (HR: 4.54, 95% CI: 1.07–19.28).

**Conclusion:**

ERCP in patients with PSC without cholangitis is likely to require further endoscopic treatment and may be associated with poor prognosis.

**Graphical Abstract:**

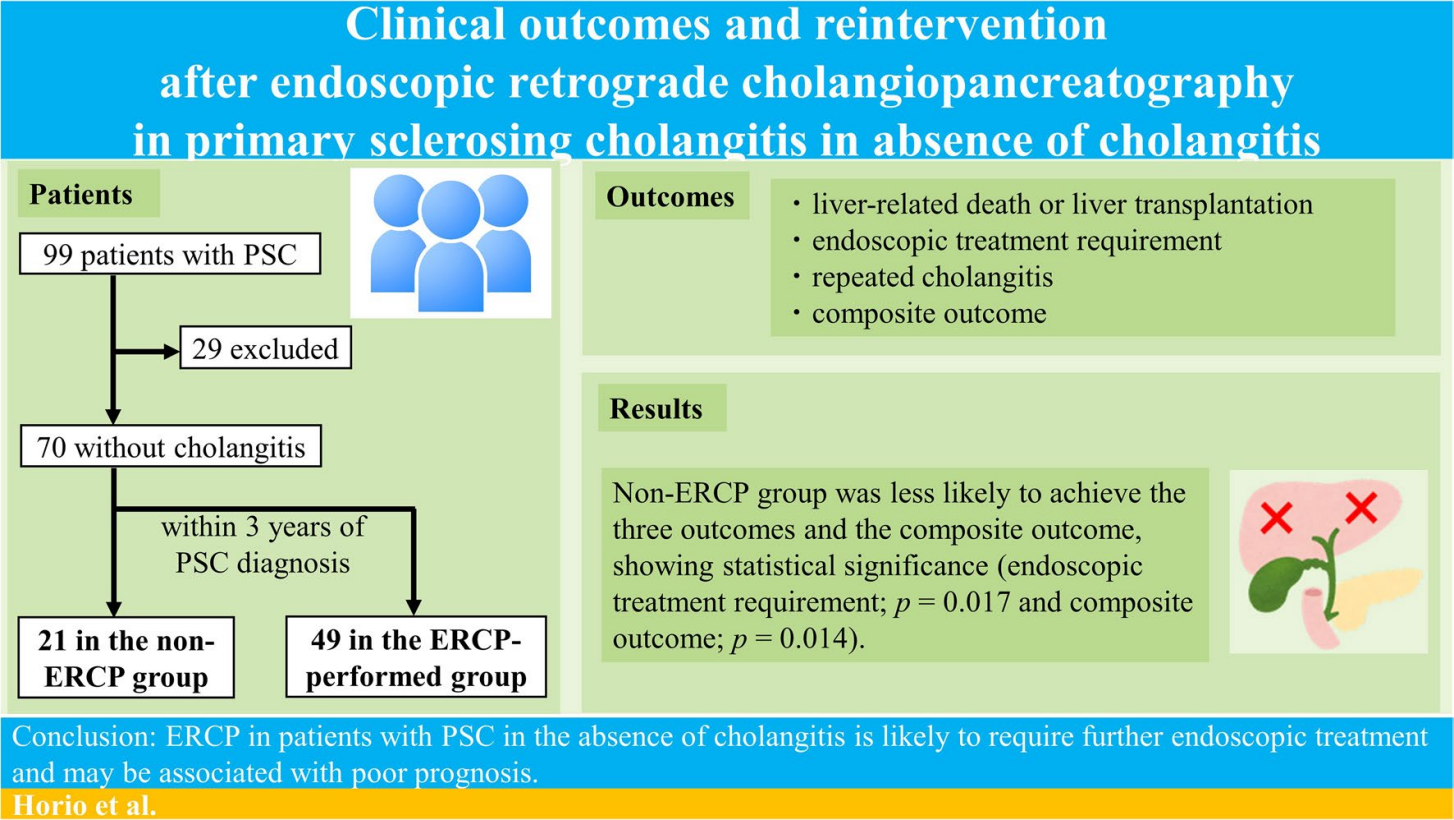

**Supplementary Information:**

The online version contains supplementary material available at 10.1007/s12664-024-01630-1.

## Introduction

Primary sclerosing cholangitis (PSC) is a progressive chronic intrahepatic biliary stasis with an unknown etiology, but it is reportedly caused by fibrous narrowing of the intrahepatic and extrahepatic bile ducts [1, 2]. The natural history of PSC is complex, with patterns ranging from little or no disease progression after a year or more to rapid disease deterioration. In general, however, PSC progresses gradually, with repeated episodes of worsening and improvement of hepatobiliary enzymes and symptom resolution and recurrence, eventually leading to cirrhosis and liver failure [3]. Currently, it has no effective drug therapy to improve prognosis and liver transplantation is its only definitive treatment [4].

For PSC diagnosis, magnetic resonance cholangiopancreatography (MRCP) is the first imaging modality of choice; conversely, endoscopic retrograde cholangiopancreatography (ERCP) is not recommended because ERCP is associated with serious complications [5–7]. However, given that patients with PSC have a risk of developing cholangiocarcinoma and one-third of cholangiocarcinoma cases are detected within one year of PSC diagnosis, ERCP may be performed primarily to check for cholangiocarcinoma even without cholangitis [8, 9].

Meanwhile, ERCP may lead to complications such as pancreatitis and cholangitis. Particularly for patients with PSC, ERCP can cause repeated retrograde cholangitis in the narrowed peripheral bile ducts; this complication can greatly impair patients’ quality of life and may worsen their prognosis [10]. Prognostic factors of PSC include age, serum bilirubin, serum albumin and Child-Pugh classification [11–14]; however, the prognostic impact of ERCP on patients without cholangitis has not been reported.

Hence, this study aimed at determining the prognostic impact of ERCP on patients with PSC without cholangitis. We also investigated the diagnosis and prognosis of cholangiocarcinoma in patients with PSC in association with ERCP conducted in the absence of cholangitis.

## Methods

### Patients

This single-center retrospective study included all patients with PSC who had regular visits to Chiba University Hospital between June 1991 and June 2022. The diagnosis of PSC was made according to the Mayo Clinic diagnostic criteria [15].

### Data collections

The data was collected from medical charts and endoscopic or radiologic images and reports. We collected the following data obtained during PSC diagnosis: age, sex, presence of cholangitis requiring endoscopic treatments, serum laboratory values, including albumin (g/dL), bilirubin (mg/dL), aspartate aminotransferase (AST) (U/L), alkaline phosphatase (ALP) (× upper limit of normal [ULN]), immunoglobulin (IgG) (mg/dL) and IgG4 (mg/dL) and carbohydrate antigen 19–9 (CA19-9) (U/mL), as well as cholangiographic findings. The following data acquired during follow-up was also collected: the time, indication and findings of ERCP, presence of inflammatory bowel disease (IBD), occurrence of cholangitis and cholangiocarcinoma and records of endoscopic treatments. Furthermore, cholangiographic findings were classified as intrahepatic, extrahepatic or both, according to ERCP and/or MRCP findings. ERCP performance for patients without cholangitis was counted within three years after PSC diagnosis, considering that some patients had time gaps between PSC diagnosis and referral to our hospital. ERCP indications for patients without cholangitis were classified according to the presence or absence of at least one of the following five criteria of the guidelines, excluding recurrent reflux cholangitis [5]: new or worsening pruritus, unexplained weight loss, worsening serum liver function test results, elevated serum CA19-9 and progressive bile duct dilatation.

Liver-related death or liver transplantation, endoscopic treatment requirement and repeated cholangitis were collected as outcome information. Endoscopic treatments included bile duct stenting, balloon dilation and lithotripsy. A recurrent cholangitis requiring endoscopic treatments at least once a month or periodic stent replacement indicated repeated cholangitis. In addition, these three outcomes were collectively called and analyzed as the composite outcome.

### Statistical analysis

The continuous variables in the table are presented as median values with interquartile ranges. Patient groups were compared using the Mann-Whitney *U*-test for continuous variables and Fisher's exact test or Pearson's Chi-square test for categorical variables. We used Kaplan-Meier survival curves for calculating the overall survival for each outcome and log-rank test for comparing survival rates between the non-ERCP and ERCP-performed groups. In addition, significant factors contributing to each outcome (liver-related death or liver transplantation, endoscopic treatment requirement or repeated cholangitis) and the composite outcome were identified using a Cox proportional hazards regression model. Evaluated variables were sex, age, presence of jaundice or cholangitis requiring endoscopic treatments, serum laboratory values (albumin, bilirubin, AST, ALP and IgG4), cholangiographic findings, presence of IBD and performance of ERCP or not in the absence of cholangitis. Significant factors in the univariate analysis were incorporated in the multivariate analysis as explanatory factors. A *p* value of < 0.05 was considered significant. All statistical data was analyzed using Statistical Package for the Social Sciences (SPSS) version 29.0 (IBM, Chicago, IL, USA).

### Ethical considerations

The study conformed to the principles of the Declaration of Helsinki and current ethical regulations, with approval from the institutional ethics committee (M10529). Written informed consent from patients was waived because data was analyzed in a retrospective, anonymized manner.

## Results

### Patient characteristics

**Fig. 1:**
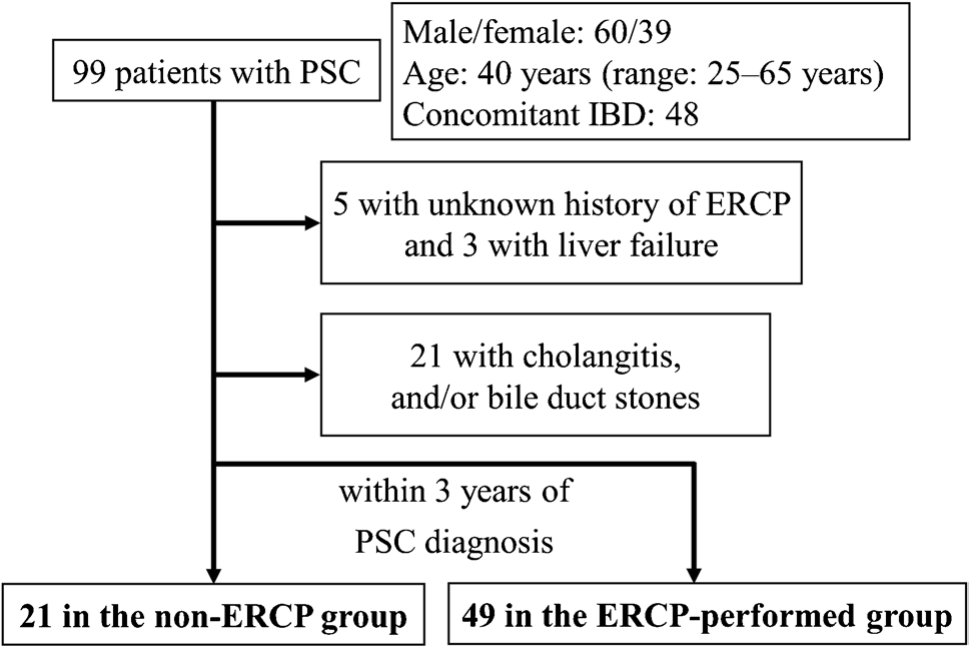
Flowchart of the study population. Those who underwent endoscopic retrograde cholangiopancreatography (ERCP) within three years after PSC diagnosis without cholangitis or bile duct stones were defined as the “ERCP-performed” group (*n* = 49) and those who did not were defined as the “non-ERCP” group (*n* = 21). *IBD* inflammatory bowel disease, *PSC* primary sclerosing cholangitis

We analyzed 99 patients with PSC who regularly visited our hospital during the study period (Fig. 1). Among them, 60 (61%) were male and the median age was 40 years (range: 25–65 years).

To examine the prognostic impact of ERCP on patients with PSC without cholangitis, we compared the outcomes between the ERCP-performed group and the non-ERCP group. Of the 99 patients, five had an unknown history of ERCP and three already had liver failure at diagnosis, thereby excluded. Moreover, 21 patients who underwent ERCP within three years after diagnosis due to symptoms such as jaundice, cholangitis and/or bile duct stones were excluded. Ultimately, 70 patients remained for the analysis, with 21 in the non-ERCP group and 49 in the ERCP-performed group (Fig. 1). Patients with both groups were followed-up mainly with regular MRCP every year and ERCP was performed when patients fulfilled the guidelines’ criteria. In our study, the median observation period was 7.2 years (range: 2.6–10.8 years).

### Prognostic impact of ERCP on patients with PSC without cholangitis

Table 1 summarizes the characteristics and outcomes of the non-ERCP group vs. the ERCP-performed group. The proportion of males was higher in the non-ERCP group (81% vs. 47%, *p* = 0.008), while the age was higher in the ERCP-performed group (28 years vs. 48 years, *p* = 0.001). The laboratory values, cholangiographic findings and the presence of inflammatory bowel disease (IBD) showed no significant differences between these groups. There was no significant difference in the median bilirubin values between the two groups (0.8 mg/dL vs. 0.8 mg/dL, *p* = 0.885). In the ERCP-performed group, six (12%) patients had one of the indications for ERCP according to the criteria of the guidelines; three had worsening serum liver function test results, two had progressive bile duct dilatation and one had unexplained weight loss. Meanwhile, the others (43/49: 88%) did not have any indications and underwent ERCP for the purpose of screening of cholangiocarcinoma. In terms of the outcomes, the ERCP-performed group was significantly more likely to have any of the three clinical outcomes (liver-related death or liver transplantation, endoscopic treatment requirement and repeated cholangitis) than the non-ERCP group (liver-related death or liver transplantation: 18% vs. 0%, *p* = 0.049; endoscopic treatment requirement: 51% vs. 10%, *p* = 0.001; and repeated cholangitis: 27% vs. 0%, *p* = 0.007).

**Table 1:** Comparison of non-endoscopic retrograde cholangiopancreatography (ERCP) and ERCP-performed groups

Variables	Non-ERCP (*n* = 21)	ERCP-performed (*n* = 49)	*p*
Sex, male:female (male %)	17:4 (81)	23:26 (47)	0.008
Age, years	28 (17–40)	48 (31–68)	0.001
Jaundice or cholangitis (%)	0 (0)	0 (0)	-
Albumin, g/dL	4.0 (3.5–4.5)	4.1 (3.9–4.4)	0.458
Bilirubin, mg/dL	0.8 (0.5–1.1)	0.8 (0.5–1.0)	0.885
AST, U/L	60 (32–92)	46 (29–91)	0.550
ALP, × ULN	2.32 (1.31–4.26)	2.35 (1.47–3.76)	0.907
IgG, mg/dL	1439 (1220–2174)	1544 (1291–1927)	0.796
IgG4, mg/dL	42.2 (19.8–111.0)	33.6 (17.5–68.0)	0.397
CA19-9, U/mL	27.4 (16.3–60.6)	21.0 (9.4–41.7)	0.170
**Cholangiographic findings**			
Both	15	42	0.155
Intrahepatic	5	7	
Extrahepatic	0	0	
Presence of IBD (%)	12 (57)	21 (43)	0.273
Liver-related death or liver transplantation (%)	0 (0)	9 (18)	0.049
Endoscopic treatments (%)	2 (10)	25 (51)	0.001
Repeated cholangitis (%)	0 (0)	13 (27)	0.007
Cholangiocarcinoma (%)	2 (10)	6 (12)	1.000

*AST* aspartate aminotransferase, *ALP* alkaline phosphatase, *ULN* upper limit of normal, *IgG* immunoglobulin, *CA19-9* carbohydrate antigen 19–9, *IBD* inflammatory bowel disease, *ERCP* endoscopic retrograde cholangiopancreatography

**Fig. 2:**
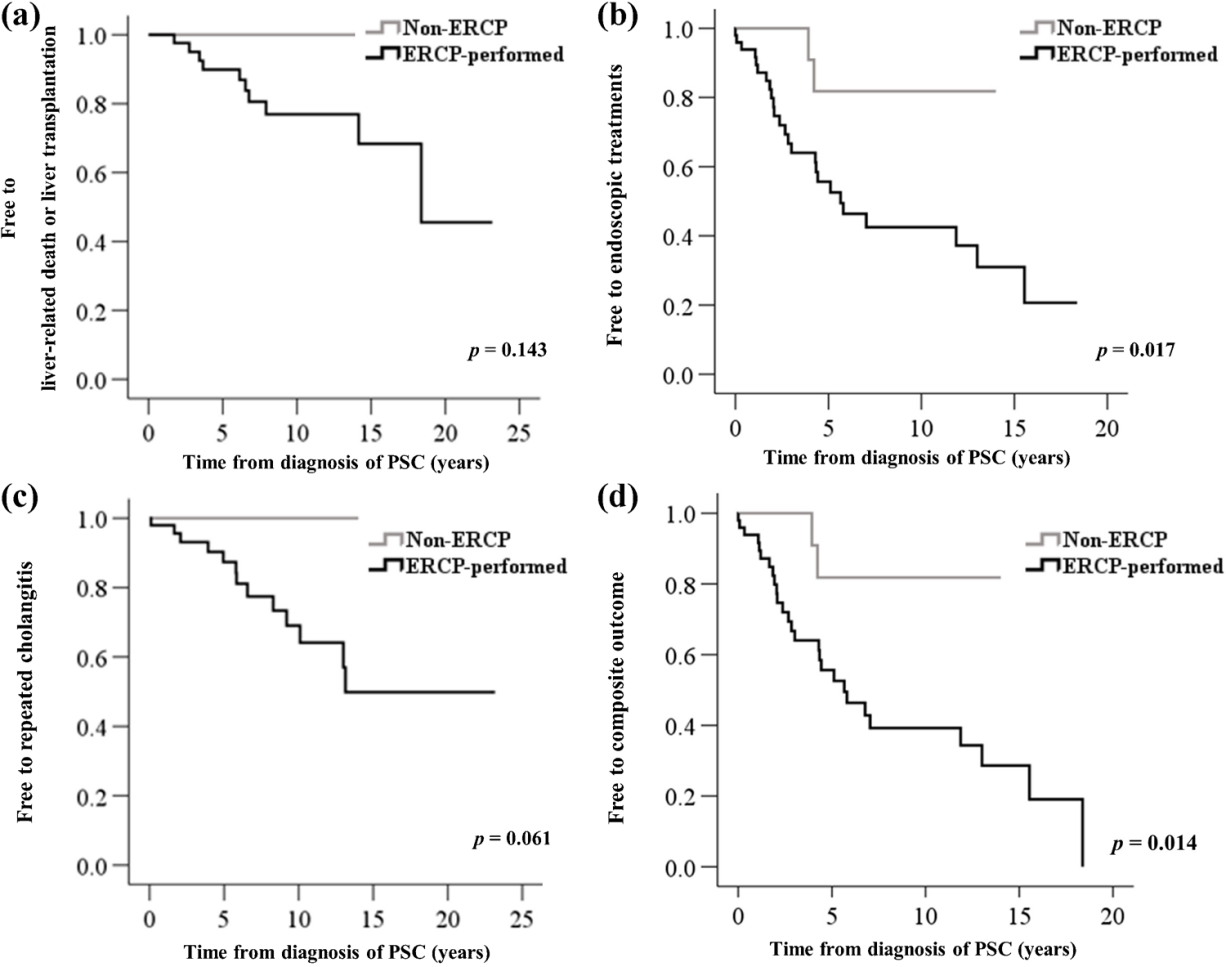
Kaplan-Meier curves of survival to liver-related death or liver transplantation (**a**), endoscopic treatments (**b**), repeated cholangitis (**c**) and the composite outcome (**d**). The non-ERCP group was less likely to achieve any outcome and composite outcome. The outcome of requiring endoscopic treatments and the composite outcome showed statistical significance

Figure 2 shows the Kaplan-Meier curves of the three outcomes and the composite outcome of the non-ERCP group vs. the ERCP-performed group. The non-ERCP group was less likely to achieve any of the three clinical outcomes and the composite outcome. Meanwhile, the endoscopic treatment requirement outcome (*p* = 0.017) and the composite outcome (*p* = 0.014) (Figs. 2b and 2d) showed statistical significance.

In addition, the analyses excluding the six cases for which ERCP indications were present according to the guidelines revealed similar results, showing statistical significance in the outcome of requiring endoscopic treatments (*p* = 0.036) and the composite outcome (*p* = 0.029).

### Prognostic factors for the outcomes

Factors contributing to the three outcomes and the composite outcome were analyzed using a Cox proportional hazards model (Tables 2–4, Supplementary Table 1). Regarding liver-related death or liver transplantation, multivariate analysis revealed that its significant predictive factors were age (≥ 40 years) (hazard ratio [HR]: 4.25, 95% confidence interval [CI]: 1.39–13.02), serum albumin (< 3.5 g/dL) (HR: 3.06, 95% CI: 1.05–8.93) and serum bilirubin (≥ 1.5 mg/dL) (HR: 3.32, 95% CI: 1.16–9.46) (Table 2). ERCP in the absence of cholangitis (HR: 4.37, 95% CI: 1.03–18.59) was the only significant predictor of endoscopic treatment requirement (Table 3). As for repeated cholangitis, the significant predictors were age (≥ 40 years) (HR: 3.24, 95% CI: 1.27–8.26) and serum bilirubin (≥ 1.5 mg/dL) (HR: 5.28, 95% CI: 2.14–13.01) (Table 4). Finally, the significant predictor of the composite outcome was ERCP in the absence of cholangitis (HR: 4.54, 95% CI: 1.07–19.28) (Supplementary Table 1).

**Table 2:** Analysis of factors predicting liver-related death or liver transplantation

Risk factors	Univariate			Multivariate		
	HR	95% CI	*p*	HR	95% CI	*p*
Sex (male)	0.89	0.38–2.13	0.801			
Age (≥ 40 years old)	3.77	1.36–10.43	0.011	4.25	1.39–13.02	0.011
Jaundice or cholangitis	1.50	0.59–3.80	0.399			
Albumin (< 3.5 g/dL)	4.20	1.49–11.88	0.007	3.06	1.05–8.93	0.041
Bilirubin (≥ 1.5 mg/dL)	3.13	1.20–8.13	0.019	3.32	1.16–9.46	0.025
AST (≥ 30 U/L)	5.33	0.71–40.1	0.104			
ALP (≥ 1.5 × ULN)	1.45	0.52–4.09	0.479			
IgG4 (≥ 135 mg/dL)	1.12	0.25–4.93	0.883			
Cholangiographic findings	0.59	0.14–2.55	0.478			
Presence of IBD	0.81	0.34–1.92	0.633			
ERCP in the absence of cholangitis	29.4	0.02–43821.41	0.365			

*AST* aspartate aminotransferase, *ALP* alkaline phosphatase, *CA19-9* carbohydrate antigen 19–9, *CI* confidence interval, *HR* hazard ratio, *IBD* inflammatory bowel disease, *IgG* immunoglobulin, *ULN* upper limit of normal,* ERCP* endoscopic retrograde cholangiopancreatography

**Table 3:** Analysis of factors predicting endoscopic treatments

Risk factors	Univariate			Multivariate		
	HR	95% CI	*p*	HR	95% CI	*p*
Sex (male)	0.84	0.46–1.54	0.571			
Age (≥ 40 years old)	1.46	0.80–2.66	0.220			
Jaundice or cholangitis	2.09	1.12–3.91	0.021	1.03	0.38–2.75	0.958
Albumin (< 3.5 g/dL)	1.14	0.44–2.95	0.780			
Bilirubin (≥ 1.5 mg/dL)	1.64	0.80–3.36	0.260			
AST (≥ 30 U/L)	1.35	0.62–2.96	0.472			
ALP (≥ 1.5 × ULN)	1.00	0.52–1.92	0.945			
IgG4 (≥ 135 mg/dL)	1.06	0.41–2.71	0.936			
Cholangiographic findings	2.70	0.65–11.17	0.182			
Presence of IBD	0.86	0.47–1.56	0.614			
ERCP in the absence of cholangitis	4.87	1.15–20.62	0.032	4.37	1.03–18.59	0.046

*ALP* alkaline phosphatase, *AST* aspartate aminotransferase, *CA19-9* carbohydrate antigen 19–9, *CI* confidence interval, *HR* hazard ratio, *IBD* inflammatory bowel disease, *IgG* immunoglobulin, *ULN* upper limit of normal, *ERCP* endoscopic retrograde cholangiopancreatography

**Table 4:** Analysis of factors predicting repeated cholangitis

Risk factors	Univariate			Multivariate		
	HR	95% CI	*p*	HR	95% CI	*p*
Sex (male)	0.98	0.43–2.20	0.957			
Age (≥ 40 years old)	2.64	1.09–6.39	0.032	3.24	1.27–8.26	0.014
Jaundice or cholangitis	2.09	0.92–4.76	0.079			
Albumin (< 3.5 g/dL)	1.33	0.39–4.62	0.650			
Bilirubin (≥ 1.5 mg/dL)	4.26	1.76–10.32	0.001	5.28	2.14–13.01	< 0.001
AST (≥ 30 U/L)	1.05	0.38–2.86	0.929			
ALP (≥ 1.5 × ULN)	1.12	0.45–2.79	0.802			
IgG4 (≥ 135 mg/dL)	1.61	0.37–6.94	0.522			
Cholangiographic findings	0.41	0.06–3.09	0.390			
Presence of IBD	0.71	0.31–1.59	0.400			
ERCP in the absence of cholangitis	29.88	0.09–9464.15	0.248			

*AST* aspartate aminotransferase, *ALP* alkaline phosphatase, *CA19-9* carbohydrate antigen 19–9, *CI* confidence interval, *HR* hazard ratio, *IBD* inflammatory bowel disease, *IgG* immunoglobulin, *ULN* upper limit of normal, *ERCP* endoscopic retrograde cholangiopancreatography

### Cases of cholangiocarcinoma

Of all 99 patients with PSC, 12 (12%) had cholangiocarcinoma (Fig. 1), with two (10%) in the non-ERCP group and six (12%) in the ERCP-performed group. In the non-ERCP group, cholangiocarcinoma was diagnosed three and five years after PSC diagnosis, respectively. In the ERCP group, three cases were diagnosed within one year after PSC diagnosis and the remaining three cases were diagnosed three, 12 and 14 years after PSC diagnosis, respectively. The occurrence of cholangiocarcinoma was not significantly different between the two groups (*p* = 1.000) (Table 1). In the ERCP-performed group, cholangiocarcinoma tended to be more likely to be observed in patients who had indications based on the criteria of the guidelines than in those who did not have (2/6: 33% vs. 4/43: 9.3%, *p* = 0.151). Of the six patients with cholangiocarcinoma in the ERCP-performed group, only one patient without cholangitis was diagnosed early with cholangiocarcinoma and may have had an improved prognosis. Conversely, the five other patients did not appear to have an improved prognosis.

## Discussion

This study demonstrated that ERCP for patients with PSC without cholangitis significantly increased the risk of requirement of endoscopic treatment and the risk of the predefined composite outcome, including liver-related death or liver transplantation and repeated cholangitis. In the multivariate analysis, ERCP, in the absence of cholangitis, was a significant predictive factor of endoscopic treatment requirement and the composite outcome and it also rarely improved the prognosis of patients with PSC who developed cholangiocarcinoma.

The current guidelines for PSC diagnosis do not recommend ERCP [5], which was once considered as the gold standard for the diagnosis of this disease [16]. Thereafter, MRCP was shown to have diagnostic accuracy similar to ERCP in the diagnosis of PSC [17]. Owing to its safety profile, MRCP has taken place of ERCP. Given that ERCP is associated with significant complications such as cholangitis and pancreatitis, it is considered to be performed only for therapeutic intervention or tissue collection [7]. However, in real clinical practice, ERCP is sometimes performed for patients with PSC without cholangitis because these patients are at risk of developing cholangiocarcinoma and one-third of cholangiocarcinoma cases is detected within one year of PSC diagnosis. During the observation period of this study, MRCP was already clinically available and in the analyzed PSC patients, MRCP was performed in almost all cases before ERCP. Therefore, it is likely that the ERCP-performed group underwent ERCP for the assessment of the presence of cholangiocarcinoma after being diagnosed with PSC through MRCP. To our knowledge, this study is the first to report the prognostic impact of ERCP on patients with PSC without cholangitis and our results indicated that this procedure could worsen the prognosis of these patients. According to our results, ERCP should not be performed for patients with PSC without cholangitis as much as possible.

As particularly observed in this study, endoscopic treatments were significantly more likely to be required in patients who underwent ERCP in the absence of cholangitis. In patients with PSC, the risk of subsequent cholangitis after ERCP seems high because of the presence of multiple stenosis of the bile duct, where intestinal bacteria are likely to remain in the peripheral once they are delivered with the procedure. In fact, the risk of repeated cholangitis was also higher in patients without cholangitis who underwent ERCP, although statistical significance was not observed. For patients with PSC with bile duct stenosis, prophylactic endoscopic treatments are sometimes required after ERCP even without subsequent cholangitis. Thus, both increase in repeated cholangitis and increase in prophylactic endoscopic treatments may contribute to the significant increase in endoscopic treatment requirement after ERCP for patients with PSC without cholangitis.

Age at diagnosis and serum albumin and bilirubin were significant risk factors of liver-related death or liver transplantation [11–14], which is often used as the ultimate prognostic outcome of PSC. Similar results were obtained in the present study and these findings suggest that PSC diagnosis at the advanced stage leads to poor prognosis. In addition, increased episodes of repeated cholangitis after ERCP for patients in the absence of cholangitis can decrease liver function, possibly resulting in increased need of liver transplantation. Meanwhile, ERCP in the absence of cholangitis did not influence the final prognosis. However, PSC is a benign disease and the duration from diagnosis to liver transplantation takes a long time, ranging from 14.5 to 21.3 years [8, 9]. In our study, the median observation period was only 7.2 years (range: 2.6–10.8 years); a longer observation period might have yielded a significant difference.

The guidelines list the following six criteria for the timing to perform ERCP: new or worsening pruritus, unexplained weight loss, worsening serum liver function test results, elevated serum CA19-9, recurrent reflux cholangitis and progressive bile duct dilatation [5]. In the present study, only six of 49 patients without cholangitis who underwent ERCP fulfilled at least one of these criteria. In addition, cholangiocarcinoma was found in two of these six patients, but only one of them may have improved prognosis because of early cholangiocarcinoma detection. Although the detection rate of cholangiocarcinoma was higher in patients who fulfilled the criteria for ERCP, the prognosis of cholangiocarcinoma rarely improved despite that ERCP performance was based on the guidelines’ criteria. Thus, the criteria of the guidelines may need to be revised from the perspective of improving the ultimate prognosis of PSC. Moreover, ERCP should not be performed without indications found in the guidelines’ criteria. However, the cohort in this study is limited by a small number of patients and it is important to note that with a larger study sample, the possibility of early detection of cholangiocarcinoma in PSC patients without cholangitis through ERCP should be considered.

In a recent large-scale multicenter study, regular imaging for the early detection of hepatobiliary malignancies in PSC has been associated with improved survival rates. In this report, the hazard ratio for mortality with a follow-up strategy, including ERCP, was 0.53 (CI: 0.37–0.75) [18], seemingly contradictory to the results of our study. However, in the discussion of the study, it is emphasized that the improved survival is attributed to the suppression of liver function decline through endoscopic treatment for advanced benign biliary strictures and no survival benefit was observed in the group undergoing regular ERCP after the diagnosis of cholangiocarcinoma or advanced dysplasia. Considering the results of our study, along with factors such as MRCP and blood test data, it is suggested that performing ERCP in PSC patients without cholangitis should be avoided when endoscopic treatment is unlikely to contribute to the improvement of liver function.

In this study, the group on which ERCP was performed was significantly older than the non-ERCP group. Our policy for patients with PSC without cholangitis was to perform ERCP for the purpose of differentiating cholangiocarcinoma in patients at the time of PSC diagnosis and the decision for its implementation had been made through discussions between physicians and patients. However, due to the need for hospitalization for ERCP, younger males were not likely to undergo ERCP. This background factor difference is considered to have resulted in the age and gender differences observed.

The strength of this study is that our hospital, as a tertiary center for PSC, has performed ERCP even for patients with PSC without cholangitis because of the high concern toward the risk of developing cholangiocarcinoma, allowing us to examine the impact of ERCP on PSC prognosis. However, because our study results have shown that ERCP for patients without cholangitis is associated not only with early complications, but also subsequent poor prognosis, ERCP for PSC without cholangitis will not be performed as often in future.

This study also has several limitations that need to be addressed. First, the study design, that is, a retrospective, single-center study, may cause unintended patient selection bias. Second, detailed information regarding ERCP procedures could not be collected. In particular, the lack of information of endoscopic sphincterotomy is a drawback because this procedure can contribute to the risk of subsequent complications [19]. Third, the age and gender differed significantly between ERCP-performed group and non-ERCP performed group and the difference may have yielded some biases. But those factors were adjusted by multivariate analyses. Both ERCP in the absence of cholangitis and patient age were identified as independent risk factors of some outcomes in this study. Finally, although Kaplan-Meier curves showed considerable differences, outcomes such as liver-related death or liver transplantation and repeated cholangitis showed no statistical significance. The small number of cases and the short duration of follow-up may have affected the results.

In conclusion, ERCP for patients with PSC without cholangitis significantly increases the risk of requiring endoscopic treatments subsequently. The risks of liver-related death and liver transplantation and repeated cholangitis are also likely to increase, although not statistically significant. In addition, ERCP rarely help improve the prognosis of cholangiocarcinoma in patients with PSC without cholangitis. The risk/benefit of ERCP for patients with PSC without cholangitis should be more carefully reconsidered.

## Supplementary Information

Below is the link to the electronic supplementary material.

Supplementary file1 (DOC 86 KB)

## Data Availability

Ensuring the confidentiality and privacy of study participants remains our foremost priority. Owing to ethical guidelines and privacy protocols, we are unable to publicly disclose the raw data utilized in our research. Nevertheless, we remain dedicated to fostering data accessibility within ethical and legal frameworks. Should there be a genuine necessity for data access, we encourage interested parties to reach out directly for potential collaboration or data sharing agreements. Each request will be meticulously evaluated, weighing privacy, ethical and legal considerations, all in pursuit of advancing knowledge while safeguarding the rights and privacy of those involved in our study.

## Declarations

### Disclaimer

The authors are solely responsible for the data and the content of the paper. In no way, the Honorary Editor-in-Chief, Editorial Board Members, the Indian Society of Gastroenterology, or the printer/publishers are responsible for the results/findings and content of this article.
