# Supplementary material for: Clinical outcomes and reintervention after endoscopic retrograde cholangiopancreatography in primary sclerosing cholangitis in absence of cholangitis
Source: Indian J Gastroenterol. 2024 Jul 12;43(5):1021–9. doi: 10.1007/s12664-024-01630-1 (PMC11464613; doi:10.1007/s12664-024-01630-1)
Supplement: Supplementary file 1 — Supplementary file1 (DOC 86 KB) [file 12664_2024_1630_MOESM1_ESM.doc]

**Supplementary Table 1. Analysis of factors predicting the composite outcome**

|  | Univariate | | |  | Multivariate | | |
| --- | --- | --- | --- | --- | --- | --- | --- |
|  | HR | 95% CI | *p* |  | HR | 95% CI | *p* |
| Sex (male) | 0.82 | 0.46–1.46 | 0.492 |  |  |  |  |
| Age (≥ 40 years old) | 1.54 | 0.86–2.75 | 0.144 |  |  |  |  |
| Jaundice or cholangitis | 2.27 | 1.24–4.14 | 0.008 |  | 0.99 | 0.37–2.64 | 0.982 |
| Albumin (< 3.5 g/dL) | 1.58 | 0.69–3.58 | 0.278 |  |  |  |  |
| Bilirubin (≥ 1.5 mg/dL) | 1.69 | 0.85–3.36 | 0.137 |  |  |  |  |
| AST (≥ 30 U/L) | 1.47 | 0.68–3.21 | 0.329 |  |  |  |  |
| ALP (≥ 1.5 × ULN) | 1.12 | 0.59–2.12 | 0.739 |  |  |  |  |
| IgG4 (≥ 135 mg/dL) | 1.10 | 0.46–2.63 | 0.825 |  |  |  |  |
| Cholangiographic findings | 2.84 | 0.69–11.7 | 0.150 |  |  |  |  |
| Presence of IBD | 0.98 | 0.55–1.74 | 0.946 |  |  |  |  |
| ERCP in the absence of cholangitis | 5.06 | 1.12–21.4 | 0.028 |  | 4.54 | 1.07–19.28 | 0.04 |

HR, hazard ratio; CI, confidence interval; AST, aspartate aminotransferase; ALP, alkaline phosphatase; ULN, upper limit of normal; IgG, immunoglobulin; CA19-9, carbohydrate antigen 19-9; IBD, inflammatory bowel disease
